# The evolutionary radiation of Arvicolinae rodents (voles and lemmings): relative contribution of nuclear and mitochondrial DNA phylogenies

**DOI:** 10.1186/1471-2148-6-80

**Published:** 2006-10-09

**Authors:** Thomas Galewski, Marie-ka Tilak, Sophie Sanchez, Pascale Chevret, Emmanuel Paradis, Emmanuel JP Douzery

**Affiliations:** 1 Laboratoire de Paléontologie, Phylogénie et Paléobiologie – CC064, Institut des Sciences de l'Evolution UMR 5554/CNRS, Université Montpellier II; Place E. Bataillon, 34 095 Montpellier Cedex 05 –, France; 2 Ecophysiologie : évolution et adaptation moléculaires, Station Biologique, Place Georges Teissier – BP 7429 680 Roscoff –, France; 3 Institut de Recherche pour le Développement, UR175 CAVIAR, GAMET – BP 5095, 361 rue Jean François Breton, 34196 Montpellier Cedex 5 –, France

## Abstract

**Background:**

Mitochondrial and nuclear genes have generally been employed for different purposes in molecular systematics, the former to resolve relationships within recently evolved groups and the latter to investigate phylogenies at a deeper level. In the case of rapid and recent evolutionary radiations, mitochondrial genes like cytochrome *b *(CYB) are often inefficient for resolving phylogenetic relationships. One of the best examples is illustrated by Arvicolinae rodents (Rodentia; Muridae), the most impressive mammalian radiation of the Northern Hemisphere which produced voles, lemmings and muskrats. Here, we compare the relative contribution of a nuclear marker – the exon 10 of the growth hormone receptor (GHR) gene – to the one of the mitochondrial CYB for inferring phylogenetic relationships among the major lineages of arvicoline rodents.

**Results:**

The analysis of GHR sequences improves the overall resolution of the Arvicolinae phylogeny. Our results show that the Caucasian long-clawed vole (*Prometheomys schaposnikowi*) is one of the basalmost arvicolines, and confirm that true lemmings (*Lemmus*) and collared lemmings (*Dicrostonyx*) are not closely related as suggested by morphology. Red-backed voles (Myodini) are found as the sister-group of a clade encompassing water vole (*Arvicola*), snow vole (*Chionomys*), and meadow voles (*Microtus *and allies). Within the latter, no support is recovered for the generic recognition of *Blanfordimys*, *Lasiopodomys*, *Neodon*, and *Phaiomys *as suggested by morphology. Comparisons of parameter estimates for branch lengths, base composition, among sites rate heterogeneity, and GTR relative substitution rates indicate that CYB sequences consistently exhibit more heterogeneity among codon positions than GHR. By analyzing the contribution of each codon position to node resolution, we show that the apparent higher efficiency of GHR is due to their third positions. Although we focus on speciation events spanning the last 10 million years (Myr), CYB sequences display highly saturated codon positions contrary to the nuclear exon. Lastly, variable length bootstrap predicts a significant increase in resolution of arvicoline phylogeny through the sequencing of nuclear data in an order of magnitude three to five times greater than the size of GHR exon 10.

**Conclusion:**

Our survey provides a first resolved gene tree for Arvicolinae. The comparison of CYB and GHR phylogenetic efficiency supports recent assertions that nuclear genes are useful for resolving relationships of recently evolved animals. The superiority of nuclear exons may reside both in (i) less heterogeneity among sites, and (ii) the presence of highly informative sites in third codon positions, that evolve rapidly enough to accumulate synapomorphies, but slow enough to avoid substitutional saturation.

## Background

During the last decade, molecular phylogenetics based on the comparative analysis of markers from mitochondrial and nuclear genomes allowed for the revision of mammalian systematics [1,2]. Sequencing efforts initially focused on mitochondrial DNA (mtDNA) due to the availability of conserved primers, the presence of rapidly evolving sites, and a reduced effective population size inducing the rapid fixation of variants between subsequent speciation events. Some protein-coding genes such as cytochrome *b *[CYB] have emerged as central tools in investigating intraspecific [e.g. [3-6]] to ordinal-level [e.g. [7-9,6]] evolutionary relationships. To date (January 2006), ca. 5,000 complete CYB entries are available for mammals in public data bases, and furthermore, another mitochondrial gene – the cytochrome *c *oxidase I – has been proposed as the marker for animal DNA barcoding [10]. However, the strong nucleotide saturation encountered at third codon positions as well as a high sensitivity to taxon sampling soon caused the questioning of the utility of mitochondrial protein-coding genes in general, and CYB in particular for resolving deep phylogenies [11,12]. Owing to their different evolutionary patterns – a less biased base composition and lower saturation than mitochondrial genes [13,14] – nuclear genes have represented a reasonable alternative to mtDNA for reconstructing deep-level mammalian phylogenies [[15-21]; but see [22]]. By providing complementary information, mitochondrial and nuclear genes are thus generally employed at different levels, low-level phylogeny and taxonomy for the former, and deeper-level for the latter.

Several mtDNA-based studies of mammalian systematics have shed light on the difficulties experienced in using CYB to resolve phylogenies at lower taxonomic levels. Several works at the family level recovered multifurcations among genera or even species, which led to the conclusion of rapid, near-simultaneous divergences of multiple lineages (= star-phylogeny), without any time left for synapomorphies to accumulate in mtDNA. This interpretation has been put forward for some speciose clades of rodents, such as Ctenomyidae [23], Echimyidae [24], or Sigmodontinae [25]. The star-phylogeny hypothesis was also supported by the lack of resolution for relationships among genera, contrasting with well-defined nodes above and below multifurcations [23,24,26]. However, an alternative hypothesis suggests that CYB sequences have undergone substitution saturation throughout the course of speciation events, leading to a loss of the original phylogenetic signal, and producing a soft-polytomy [27]. In this case, the use of slower-evolving nuclear DNA (nuDNA) might represent a better choice for resolving such recent radiations. Supporting this hypothesis, sequences from such markers, either exons or introns, have recently improved among-families [28-30], or even among-genera [31,32] phylogenetics of spiny rats, squirrels, whales, weasels, and spiral-horn antelopes.

In the present study, we focused on the evolutionary history of voles, lemmings, and muskrats. These animals belong to the Arvicolinae, one of the six Cricetidae subfamilies of the highly-diverse Muroidea, which encompasses one third of all rodent species. The Arvicolinae represent themselves one of the most impressive placental radiations in the Northern Hemisphere, consisting of 151 species and 28 genera [33]. As stated by [33], the explosiveness and recency of arvicoline evolution can be dramatically highlighted by the more than 60 species of *Microtus *and the inconsistency of their systematic treatment. Phylogenetic reconstructions based on mitochondrial sequences (CYB and NADH-dehydrogenase 4 [ND4]) provided unresolved topologies at two different levels: (1) among arvicoline genera, and (2) among species of *Microtus *[34-36]. These results reinforced observations previously made by paleontologists [37,38] who concluded that two successive pulses of speciation occurred during the evolutionary course of arvicoline rodents over the last 10 million years (Myr).

Following the encouraging results from recent studies [28-32], we thus compared the relative contribution of a nuclear marker – exon 10 of the growth hormone receptor (GHR) gene – to one of the mitochondrial CYB for inferring phylogenetic relationships among major Arvicolinae lineages. We performed analyses in a probability framework because (i) maximum likelihood (ML) and Bayesian methods are indeed based upon explicit models of sequence evolution, (ii) they allow the definition of independent models to reflect the contrasting substitution patterns among the three codon positions of GHR and CYB, and (iii) they have proven to be robust to a number of systematic biases during phylogenetic reconstruction [39-41]. In addition to such inter-sites comparisons, we evaluated the amount of saturation at each codon position. Using the variable-length bootstrap method, we also estimated the amount of sites required by mitochondrial, CYB-like, and nuclear, GHR-like data to resolve recent and deeper nodes of the evolution of voles and lemmings.

## Results and discussion

### 1. Mitochondrial and nuclear phylogenies of the Arvicolinae

#### (i) Contribution of CYB

**Figure 1:**
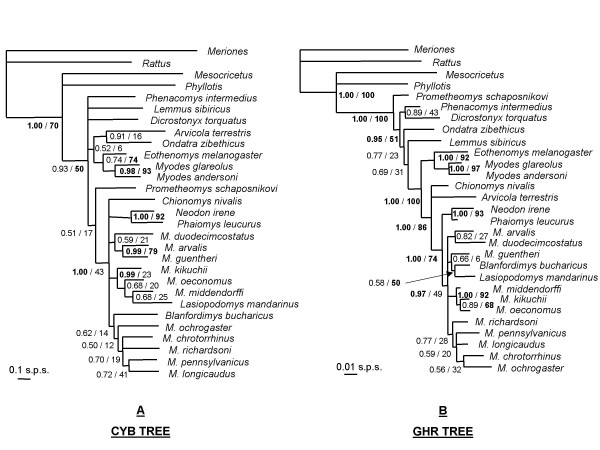
**Maximum posterior probability trees reconstructed from the mitochondrial CYB (left, A) and nuclear GHR (right, B) sequences**. Two reliability indices are given on nodes: the Bayesian posterior probabilities/the maximum likelihood bootstrap percentages. Note the difference of scale (expressed as substitutions per sites [s.p.s.]).

Both Bayesian and ML DNA analyses provide poorly resolved phylogenies, with most of arvicoline genera and most of *Microtus *species arising from a polytomy (Figure 1A). Only three nodes benefit from strong support (BP_ML _> 70 and PP > 0.95), four are moderately supported (BP_ML _> 50 or PP > 0.95), whereas the 18 remaining nodes do not receive significant support. The three clades unambiguously identified (Figure 1A) involve: (i) both *Myodes *species included in our analysis: *M. glareolus *and *M. andersoni*; (ii) *Neodon irene *+ *Phaiomys leucurus*; and (iii) *Microtus (Microtus*) *arvalis *+ *M*. (*Microtus*) *guentheri*. At the amino acids level, only 12 % of the CYB sites are parsimony informative among arvicolines, and, as already observed by [34], the corresponding topology is not resolved (a single node is supported by BP_ML _> 70).

#### (ii) Contribution of GHR exon 10

Branching patterns recovered with ML and Bayesian methods are identical. GHR sequences provide much more resolved topologies (Figure 1B) than those based upon CYB sequences, with eight strongly and four moderately supported nodes. All clades previously identified in the CYB tree are recovered with higher support, excepted for the sister-clade relationship between *Microtus arvalis *and *M. guentheri *not found in GHR trees. A better resolution involves all taxonomic levels from internal suprageneric relationships to intra-*Microtus *nodes (Figure 1B). New, well-supported clades include: (i) the monophyly of the subgenus *Alexandromys *(*Microtus kikuchii, M. oeconomus*, and *M. middendorffi*); (ii) the monophyly of a *Microtus sensu lato *clade (*Neodon, Phaiomys, Blanfordimys, Lasiopodomys*, and *Microtus*) but excluding *Chionomys*; (iii) the grouping of *Arvicola + Chionomys + Microtus s.l*.; (iv) the association of *Eothenomys *with *Myodes *(= Myodini); and (v) a sister-clade relationship between Myodini and Arvicolini. At the amino acids level, 16 % of the GHR sites are parsimony informative among arvicolines, and the corresponding topology is poorly resolved (three nodes are supported by BP_ML _> 70).

We also scanned the GHR alignment for potential indel signatures. A 3-bp deletion at position 1,925 of the GHR sequence of *Rattus norvegicus *(Accession number NM_017094) is diagnostic for the monophyly of Arvicolinae. A 3-bp in-frame insertion – a direct AGC repeat – at position 1,778 of *Rattus norvegicus *(Accession number NM_017094) is shared by *Neodon *+ *Phaiomys*, and independently by *Blanfordimys *+ *Lasiopodomys*.

Independent paleontological and molecular studies respectively estimated that divergence times among some arvicoline genera occurred 3–5 Myr ago (Mya) [37] to 5–9 Mya [30,34,42]. The adaptive radiation of *Microtus *has been dated to approximately 2 Myr by most paleontologists [37,43] but there is molecular evidence for splits between 2.6 to 4.4 Mya [34]. The GHR topology is here more resolved than the CYB one for intergeneric speciation events, corresponding to mean uncorrected pairwise divergences of 3.8 % ± 0.9 (GHR) versus 14.2 % ± 1.6 (CYB). For the 2–5 Myr old *Microtus *radiation, corresponding to divergences of 2.2% ± 0.6 (GHR) and 11.7 % ± 1.2 (CYB), the GHR topology is at least as resolved as the CYB one. These results emphasize previous conclusions [44] that highlighted the superiority of nuclear genes over mitochondrial genes even for divergences spanning the last 5–10 Myr.

#### (iii) Combination of CYB and GHR genes

**Figure 2:**
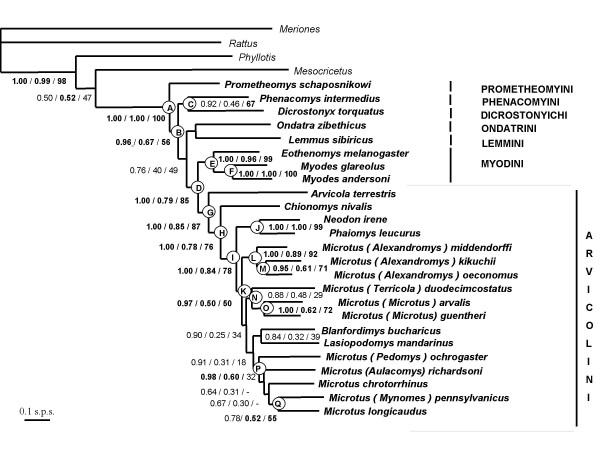
**Maximum posterior probability tree reconstructed from the combination of the mitochondrial CYB and nuclear GHR sequences**. Three reliability indices are given on nodes: the Bayesian posterior probabilities/the bootstrapped Bayesian posterior probabilities/the maximum likelihood percentages. Letters (from A to Q) refer to nodes recovered both in ML and Bayesian inferences (see Table 2).

Bayesian and ML analyses of the concatenation of mitochondrial and nuclear data sets provide highly congruent and globally well-resolved topologies, very similar to the ones based upon GHR alone. Nevertheless, increased support values are generally recorded. Thus, nine nodes are strongly supported and six are moderately supported. The newly recovered associations involve nodes labeled H, B, C, P, and Q (Figure 2): (i) *Chionomys *sister-group of *Microtus s.l*. with *Arvicola *placed at the base of Arvicolini; (ii) *Prometheomys *emerging first within Arvicolinae; (iii) *Dicrostonyx *+ *Phenacomys*; (iv) a clade, only supported in the Bayesian analysis (PP = 0.97; BP_bay _= 0.60), and uniting five *Microtus *species: *M. (Pedomys) ochrogaster, M. (Aulacomys) richardsoni, M. chrotorrhinus, M. (Mynomes) pennsylvanicus*, and *M. longicaudus*; (v) *Microtus (Mynomes) pennsylvanicus *sister-group of *Microtus longicaudus*.

Clades moderately to strongly supported in the GHR tree data benefit from similar support values after the combination of GHR and CYB sequences. Actually, a substantial increase of support is found for nodes defining the basal position of *Prometheomys*, and the monophyly of *Microtus s.l*. is reinforced. On the opposite, the sister-clade relationship recovered between Myodini and Arvicolini is less supported in the combined tree than in the GHR one. Expectedly, the *M. arvalis *+ *M. guentheri *clade, recovered by CYB data, is similarly supported in the combination.

To summarize, the combination of CYB and GHR sequences provides a phylogeny where two-third of the nodes benefit from a medium to high support (Figure 2). The addition of nuclear sequences also allows the division of a single, multi-taxa polytomy into two noticeably smaller multifurcations. The first includes Arvicolini, *Ondatra*, *Lemmus*, *Dicrostonyx*, and *Phenacomys*, although the latter two genera are consistently found as sister-group across the different reconstructions performed. The second multifurcation includes phylogenetic relationships among *Microtus s.l*. subclades with evidence for a deeper divergence of the *Neodon + Phaiomys *lineage.

### 2. Systematics of voles, lemmings, and muskrats

#### (i) Interrelationships of major genera

For the first time, and contrary to phylogenies based on mitochondrial data alone [34], the use of a nuclear DNA exon sheds light on the systematics of arvicoline rodents. The validation of the Arvicolini tribe is one of the most striking results. The molecular evidence for a clade including *Arvicola*, *Chionomys*, and *Microtus s.l*. (Figure 2) – with the water vole emerging at first – agrees with paleontology. Actually, the genera *Microtus *and *Chionomys *are supposed to be linked to *Allophaiomys *which represents an early split from the lineage *Mimomys *that led to *Arvicola *[37]. Our results concur with other research [45,46] that red-backed voles form a complex of closely related species as suggested by the strong support recovered for the grouping of *Myodes*, (i.e., *Clethrionomys *and *Phaulomys sensu *[47]) with *Eothenomys*.

In addition, a first molecular evidence is here provided for the phylogenetic position of enigmatic genera suspected to have differentiated early within the arvicoline radiation. The long-clawed mole-vole, *Prometheomys*, currently localised in the Caucasus Mountains, was considered as a relic of an archaic lineage formerly widespread throughout most of Eurasia [33,48-51]. This view is corroborated by its first emergence in the GHR and combined phylogenetic trees (Figures 1B and 2). The collared lemming, *Dicrostonyx *as well as the heather vole, *Phenacomys*, exhibit plesiomorphic morphological traits which have compromised their systematic affiliation to other arvicolines. *Dicrostonyx *was initially grouped with true lemmings whereas *Phenacomys *was included in either Arvicolini or Myodini [33]. By obtaining a moderately supported sister-taxa relationship between *Dicrostonyx *and *Phenacomys*, our study is in accordance with previous result from the analysis of highly repetitive DNA (LINE-1) elements [52]. Although unexpected, this clade might find a biogeographical explanation as these two taxa are primarily known – from a palaeontological viewpoint – in the Arctic region of Eurasia and North America [37,53,54].

To summarize our results from a taxonomic standpoint, the phylogenetic relationships discussed above agree with the tribal recognition of Lemmini (*Lemmus*), Ondatrini (*Ondatra*), Dicrostonychini (*Dicrostonyx*), Phenacomyini (*Phenacomys*), Prometheomyini (*Prometheomys*), Myodini (*Myodes *and *Eothenomys*) and Arvicolini (*Arvicola*, *Chionomys*, *Microtus*, *Neodon*, *Phaiomys*, *Blanfordimys*, and *Lasiopodomys*) [33].

#### (ii) Systematics of Microtus

Thanks to the sampling of new taxa and nuclear DNA data, our phylograms helped to test the accuracy of systematic treatment of *Microtus *taxa and close genera. Some taxa – *Blanfordimys, Chionomys, Lasiopodomys, Neodon, Phaiomys *– have indeed been split from *Microtus *because they retain distinctive and plesiomorphic morphological/caryological traits [33]. However, except for *Chionomys *(snow voles), other taxa are interspersed among "true" *Microtus *lineages in our nuclear or combined topologies, giving no support to their generic recognition.

Actually, three lineages are clearly identified within *Microtus*. Firstly, strong evidence is provided for a *Neodon *+ *Phaiomys *subgenera clade, contradicting former studies [33] which, using molar and other external and cranial contrasts, rejected any close phylogenetic affinity between them. Secondly, *Microtus kikuchii, M. oeconomus*, and *M. middendorffi *form a well-supported association. Independent source of phylogenetic data – here nuclear GHR sequences – thus confirm the "Asian lineage" recently identified on the basis of mitochondrial phylogenies and chromosomal data [35,36,55], as well as the monophyly of the subgenus *Alexandromys*, as redefined in [33]. The five nearctic species included in our analysis and representatives of various subgenera (*Aulacomys, Mynomes, Pedomys*, as well as *Microtus longicaudus *and *M. chrotorrhinus *whose systematic status is unclear) are associated with some support in the combined analysis. The recognition of an American clade within *Microtus *strengthens previous results from biochemical analyses [37] and mitochondrial phylogenies [35,36]. Third, some support for a *Terricola *+ *Microtus *clade is recovered, suggesting close phylogenetic affinities among Western Palaearctic taxa, and reinforcing the conclusions of [36].

### 3. Compared molecular evolution of CYB and GHR in arvicolines

#### (i) Characteristics of substitution patterns

Nucleotide substitutions appear to accumulate in CYB and GHR according to contrasted patterns. CYB evolves at a high rate, 11.6 times faster than GHR does as indicated by the total branch length of ML phylograms. Moreover, as expected for a mitochondrial protein-coding gene [7], Table 1 indicates that CYB: (i) has a base composition that greatly deviates from uniformity due to an important deficit of G (< 10%) and an excess of C and A (ca. 35%) ; (ii) is characterized by strong biases in relative substitution rates, with an excess of transitions over transversions, notably due to a higher frequency of C-T changes ; (iii) shows a stronger rate heterogeneity among sites, with a Γ shape parameter (α = 0.17) lower than the one of GHR (α = 0.48). These patterns reflect the fact that most of CYB substitutions occur on third codon positions and are generally synonymous.

**Table 1:** Bayesian estimates of DNA substitution model parameters for CYB, GHR, and their codon positions.

	**CYT B**			**GHR**		
	
	**1**	**2**	**3**	**1**	**2**	**3**
**Sites**	381	381	381	307	307	307

**% A**	**36.0** *33.7–38.4*			**28.2** *25.6–31.0*		
	
	**30.5**	**20.9**	**42.7**	**27.7**	**34.3**	**20.2**
	*26.4–34.6*	*17.1–25.0*	*38.5–46.4*	*23.2–32.3*	*29.5–39.2*	*16.5–24.2*

**% C**	**33.3** *31.4–35.3*			**27.4** *24.8–30.1*		
	
	**28.8**	**25.7**	**33.6**	**21.8**	**30.4**	**30.9**
	*24.9–32.9*	*21.8–29.9*	*30.7–36.4*	*17.8–26.1*	*25.9–35.1*	*26.7–35.3*

**% G**	**9.2** *7.8–10.6*			**23.0** *20.5–25.1*		
	
	**22.2**	**11.7**	**3.8**	**33.4**	**14.6**	**21.2**
	*18.4–26.1*	*8.8–14.9*	*3.1–4.7*	*28.7–38.4*	*10.9–18.4*	*17.4–25.3*

**% T**	**21.5** *20.0–23.0*			**21.8** *19.5–24.3*		
	
	**18.5**	**41.6**	**19.9**	**17.1**	**20.7**	**27.7**
	*15.6–21.8*	*37.1–46.3*	*17.8–22.4*	*13.6–21.2*	*16.8–24.8*	*23.5–32.1*

**r AG**	**6.8** *4.0–11.7*			**5.8** *3.5–9.3*		
	
	**4.3**	**1.0**	**22.1**	**7.3**	**17.9**	**13.5**
	*2.0–8.4*	*0.1–3.5*	*4.2–83.7*	*2.4–19.5*	*2.8–68.7*	*6.1–29.0*

**r CT**	**19.9** *11.2–37.6*			**6.5** *4.0–10.4*		
	
	**37.2**	**40.2**	**19.0**	**6.2**	**20.2**	**5.4**
	*12.3–78.2*	*7.7–93.7*	*3.8–76.0*	*2.0–16.2*	*2.9–72.0*	*2.6–10.4*

**r AC**	**0.7** *0.4–1.4*			**1.06** *0.6–1.8*		
	
	**0.6**	**0.3**	**1.1**	**2.5**	**2.7**	**1.0**
	*0.2–1.4*	*0–1.7*	*0.2–4.4*	*0.7–7.3*	*0.4–10.7*	*0.4–2.3*

**r AT**	**1.6** *0.8–3.1*			**0.9** *0.4–1.5*		
	
	**1.8**	**0.4**	**2.2**	**2.2**	**2.9**	**0.8**
	*0.6–4.0*	*0.1–1.4*	*0.4–8.4*	*0.6–6.4*	*0.2–13.0*	*0.3–1.8*

**r CG**	**0.3** *0.1–0.7*			**1.5** *0.8–2.6*		
	
	**0.2**	**14.4**	**2.4**	**2.1**	**6.4**	**1.3**
	*0–0.7*	*1.6–49.5*	*0.1–10.9*	*0.6–5.6*	*1.0–24.3*	*0.5–2.7*

**α (Γ **_8_**)**	**0.17** *0.16–0.19*			**0.48** *0.41–0.53*		
	
	**0.14**	**0.05**	**1.22**	**0.05**	**0.05**	**0.22**
	*0.12–0.17*	*0.05–0.06*	*0.96–1.54*	*0.05–0.05*	*0.05–0.06*	*0.18–0.27*

Number of sites, base composition (%A, C, G, and T), relative GTR substitution rate parameters (r AG, CT, AC, AT, CG, standardized to r GT = 1.0), and Gamma shape parameter (Γ) are given, with mean values on first sublines, and 95% credibility intervals, italicized on second sublines. Parameter estimates are also measured for each codon position, and are recapitulated in columns 1 (first), 2 (second), and 3 (third positions).

#### (ii) Contrasted substitution patterns among codon positions

As generally pointed out for nuclear genes relative to mitochondrial ones, GHR exhibited a less contrasted substitution pattern during its evolutionary course. For instance, more heterogeneity among codon partitions is observed for CYB relative to GHR for two parameter sets. (i) Evolutionary rates – as measured by total branch lengths (TBL) on ML phylograms – are highly heterogeneous among CYB codon positions, with CYB3 (TBL = 58.20) evolving respectively about 24 and 166 times faster than CYB1 (TBL = 2.39), and CYB2 (TBL = 0.35). On the contrary, evolutionary rates are moderately contrasted among GHR codon positions, with GHR3 (TBL = 1.17) evolving three times faster than GHR1 (TBL = 0.41) and GHR2 (TBL = 0.34). (ii) Base composition also appears more heterogeneous among CYB codon positions (Table 1), with 11.7% and 3.8% of G for CYB2 and CYB3 respectively. By contrast, only GHR2 shows such a low value of G (14.6%). An excess of T in CYB2 (41.4%) and A in CYB3 (43.2%) is also noticed. Moreover, all GHR codon positions pass the 1% chi-square test that compares the nucleotide composition of each sequence to the frequency distribution assumed in the ML model, whereas *Microtus oeconomus *and *M*.*middendorffi *CYB third codon positions violate it.

#### (iii) Partitioned likelihood analyses

The marked differences of base composition and evolutionary dynamics observed among CYB and GHR codon positions led us to apply the partitioned likelihood approach [56] in order to evaluate their impact on model fit (Table 2). For both CYB and GHR, the AICs for partitioned models are the lowest, confirming a gain in log-likelihood when an independent set of parameters is attributed to each codon position. Actually, AICs indicate that all sets of parameters for CYB are contrasted enough among codon positions to have an impact on model fit (Table 2). More precisely, the greater increase of log-likelihood values is due, in decreasing order of impact, to variable rates between sites (Γ), to variable evolutionary rates along branches (BL), to variable base compositions (BC), and to variable GTR substitution rates (GTR). By contrast, for GHR sequences, AICs indicate that only BC and Γ have a significant impact on model fit, whereas the incorporation of free GTR and BL parameters for each of the three codon positions induces the over-parametrization of such models (Table 2). These analyses confirm that there is more heterogeneity among CYB codon partitions than among GHR partitions.

**Table 2:** Akaike Information Criterion (AIC) sensitivity analyses about the effect of model parameters on log-likelihood gains provided by codon partitions.

	**Model of DNA evolution**					
	
	No codon partition	BC effect	GTR effect	Γ effect	BL effect	BC + GTR + Γ + BL effect
**CYB**	23,021.0	22,742.4	22,954.8	22,509.1	22,689.3	**21,901.9**
**GHR**	8,352.0	**8,306.1**	8,347.7	8,346.2	8,434.3	8,388.3

In the simplest model – without codon partition–, a single set of base composition (BC), substitution rate (GTR), among-sites rate heterogeneity (Γ), and branch length (BL) parameters is used, irrespective of the CYB and GHR codon positions. In the most complex model, one independent set of BC, GTR, Γ, and BL parameters is attributed to each codon position of either CYB or GHR. For intermediate models, the effect of giving a set of either BC, GTR, Γ, or BL parameters to each codon partition is measured through the AIC (= -2 × log-likelihood of the reference topology [Figure 2] + 2 × number of free parameters). The best, i.e., lowest, AIC are in bold for each gene.

### 4. Identification of promising nucleotide sites for arvicoline phylogenetics

Although some studies have elucidated the limited phylogenetic utility of individual nuclear genes at low taxonomic level because of their low variability [29,32,57], we provide here a well-resolved GHR tree among Arvicolinae. We will now explore why GHR performs better than CYB for deeper nodes – the inter-generic arvicoline radiation –, and also provides increased support to more recent nodes – the *Microtus *sublineages.

#### (i) Localization of the phylogenetic signal among codon positions

The analysis of the contribution of each codon partition to node resolution reveals that GHR3 holds the most informative sites (Table 3). GHR3 sites recover most of the nodes with strong support, especially within the intergeneric radiation. Although its phylogenetic signal generally appears slightly lower for the last 2 Myr divergences (e.g., intra-*Microtus *nodes), globally, GHR3 remains the most informative partition. GHR1 and CYB3 contribute much less than GHR3 to overall resolution but hold some support (BP > 40) for ca. one-third of the nodes. On the contrary, other partitions – CYB1 and, especially, CYB2 and GHR2 – are not informative for intra-Arvicolinae relationships as reflected by exceedingly low bootstrap scores (BP < 28: Table 2). With respect to nodes that are revealed or are more strongly supported by the combination of CYB and GHR (e.g. nodes H and P), we notice that no partition is superior in resolution ability with a weak signal being shared by all codon positions.

**Table 3:** Maximum likelihood bootstrap support for a selection of nodes, computed according to codon positions 1, 2, or 3 of CYB and GHR.

**Nodes**	**CYB 1**	**CYB 2**	**CYB 3**	**GHR 1**	**GHR 2**	**GHR 3**
Intergeneric relationships						
*Phyllotis *+ *Mesocricetus *+ Arvicolinae	**70**	32	*	**63**	40	**66**
A	*	**63**	*	**88**	35	**99**
B	*	*	*	44	*	21
C	28	*	11	*	28	34
D	*	*	*	*	14	**90**
E	*	*	45	*	*	**91**
F	**55**	*	**50**	43	*	**98**
G	*	*	*	20	*	**87**
H	*	20	33	20	*	*
I	*	22	33	*	*	**81**

Intra-"*Microtus*" relationships						
J	14	18	**79**	**52**	22	**92**
K	*	*	*	*	*	41
L	8	*	47	**55**	*	**82**
M	40	10	14	*	28	34
N	*	*	11	*	*	15
O	26	*	42	*	*	31
P	*	*	*	*	*	27
Q	44	*	*	*	*	29

Node lettering refers to Figure 2. Stars correspond to bootstrap scores inferior to 5% for the corresponding node under the codon partition analyzed.

#### (ii) Substitution saturation through time

**Figure 3:**
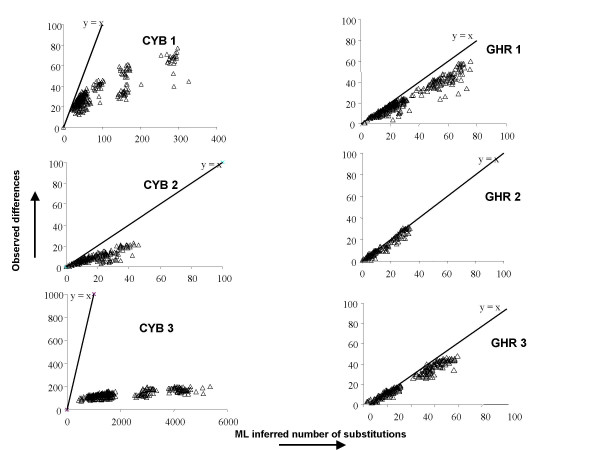
**Saturation plots of the number of observed differences as a function of the numbers of inferred substitutions for each pair of sequences at each codon position of CYB and GHR genes**. The Y = X straight line corresponds to the situation where there is no homoplasy detected in the data. Note that the scale of the X-axis is four-fold the scale of the other partitions for CYB1, and 60-fold for CYB3. The scale of the Y-axis is 10-fold the scale of the other partitions for CYB3.

The lack of phylogenetic signal at high taxon level often recorded by mitochondrial sequences has been considered as a direct consequence of substitution saturation [e.g. [27,58]]. We thus evaluated the saturation level of each codon partition by plotting the pairwise observed substitutions between the 28 sequences as a function of the pairwise number of substitutions inferred on the ML tree (Figure 3). Under this graphical representation, CYB3 appears extremely saturated (slope S = 0.02), suggesting that high levels of nucleotidic saturation may be reached for speciation events more recent than 5–10 Ma. This trend is perhaps here exaggerated by the fast evolutionary rate of muroid rodent sequences [44]. Multiple substitutions also occur at CYB1 (S = 0.17) whereas analyses for CYB2 (S = 0.47) and all GHR codon positions (0.69 < S < 0.99) do not reveal significant saturation as detected by ML. Codon partitions can thus be categorized into three classes corresponding to (i) highly saturated and weakly informative sites (CYB1 and CYB3); (ii) less saturated and weakly informative sites (CYB2, GHR1, and GHR2); (iii) less saturated and strongly informative sites (GHR3).

#### (iii) The efficiency of GHR third codon positions

By comparing contribution to node resolution, saturation level, and evolutionary patterns inherent to each codon partition, it becomes possible to determine the characteristics of informative versus uninformative molecular sites. We thus realize that GHR3 differed from uninformative CYB2, GHR1, and GHR2 partitions by a faster evolution rate (see above, section 3). In contrast, CYB3 and CYB1 partitions are much less informative than GHR3 despite higher evolutionary rates that would have allowed the accumulation of a larger number of synapomorphies. A high substitution rate thus appears detrimental too, having been more likely to have led to saturation (Figure 3). Moreover, strong biases in base composition and GTR substitutions could also favour substitutional saturation. For instance, although CYB1 and GHR3 share similar evolutionary properties (GC levels, Γ shape, GTR rates: Table 1) – CYB1 is more affected by multiple substitutions because of either a slightly higher evolutionary rate or an excess of C-T over A-G transitions. Our study therefore suggests that third codon positions of GHR are the most suitable sites for resolving the recent evolutionary radiation of arvicoline rodents. The higher efficiency of GHR3 is a result of the trade-off in evolutionary rate, rapid enough to accumulate synapomorphies, yet slow enough to remain unaffected by saturation.

5. Perspectives: Which markers to be used for resolving the arvicoline radiation?

The resolved GHR tree is perhaps a first step in challenging the hypothesis of a hard-polytomy for Arvicolinae genera. As shown for other placental taxa – spiral-horn antelopes [32], hares [57], or bears [59] – our results suggest that phylogenetic signal has been progressively obliterated by higher mtDNA rates whereas it has persisted in at least one slower-evolving nuclear gene. However, the GHR gene tree is based on a single nuclear locus (GHR), and it does not necessarily reflect the organismal history of arvicoline rodents. Actually, the resolution of the polytomy is further complicated by the eventual incongruence of individual gene trees with species trees due to incomplete lineage sorting [60]. Consequently, the comparison of multiple gene trees is the only way to get a central tendency which could be interpreted as the species tree [61-63]. If multiple gene trees agree on a phylogenetic structure, we could then definitively dismiss the star-phylogeny hypothesis for the Arvicolinae. In addition to allowing us to make predictions on the amount of CYB/GHR data required to decipher the phylogeny of the Arvicolinae, variable length bootstrap analyses help us to identify which kind of markers should be considered for further sequencing.

#### (i) The not-so-evil mitochondrial DNA

**Figure 4:**
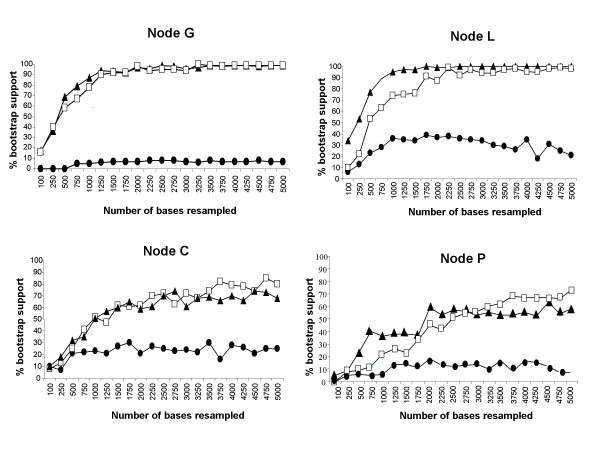
**Plots of the variable-length bootstrap percentages for CYB (filled circles), GHR (filled triangles), and combined data (open squares) for four nodes recovered in Bayesian and ML analyses (see Fig. 2)**. The X-axis is the number of sites resampled (in 250-bp increments from 0 to 5000-bp), and the Y-axis is the maximum likelihhod BP percentage (out of 100 pseudoreplicates).

Four examples of VLB curves are provided on Figure 4: two represent deeper (intergeneric) nodes, moderately (node C) to strongly (node G) supported in the GHR + CYB tree, and two others correspond to more recent (intra *Microtus*) nodes, moderately (node P) to strongly (node L) supported in the combined topology. For most nodes (including nodes C, G, L, P; Figure 4), the resampling of more CYB sites does not induce an increase of bootstrap support. Despite using a ML model for VLB analyses which better accommodates base composition bias and among-site rate heterogeneity, CYB sequences do not appear more efficient than in other studies focusing on comparable taxonomic levels [29]. Thus, the analysis of more data holding the same evolutionary properties as CYB might not bring more overall resolution.

The lack of phylogenetic signal as well as strong saturation and large among-sites heterogeneity questions the utility of CYB sequences for investigating phylogenetic relationships among taxa, like voles and lemmings, which have experienced relatively recent and rapid radiations. Actually, we concur with others [64,65] on the usefulness of CYB sequences – including third codon positions – when they are combined with less saturated sequences. Firstly, we have shown that CYB sometimes holds phylogenetic signal for specific nodes where GHR is non-informative (node O, see Results & Discussion, paragraph 1). Although our study mainly focuses on intergeneric phylogenetic relationships, we could expect that CYB contribution would increase with nodes younger than 2 Myr. Secondly, VLB curves clearly indicate that data combination (CYB + GHR) provides slightly higher bootstrap support than does GHR alone, especially when the number of resampled sites is higher than 2,000 (e.g., nodes L and P: Figure 4). Results of VLB analysis and comparisons of GHR and combined topologies show that the CYB signal does not contradict the GHR one. Moreover, the incorporation of CYB data to nuclear data does not lead to the degradation of phylogenetic signal but slightly improves it, revealing the existence of weak and initially hidden information within CYB. Because complete mitochondrial genomes include sequences undergoing various evolutionary pressures (e.g. CYB, but also COX1, or rRNAs), and are available for an increasing number of taxa, particularly among mammals, we suggest the evaluation of their ability to resolve rapid radiations at low taxonomic level [66], and a comparison of their efficiency to that of nuclear loci.

#### (ii) The virtues of nuclear DNA

As exemplified in Figure 4, GHR performs better than CYB for most nodes, irrespective of the age of the node. More generally, VLB curves indicate a higher efficiency of GHR relative to CYB sequences for nearly all nodes, i.e., they recover nodes with higher bootstrap support with a lower number of resampled sites. Even for weakly supported nodes in our initial analyses of ca. 1000 GHR sites, only 2,000 to 4,000 GHR additional sites are needed to increase BP values up to 70% or more. We may thus expect a significant improvement in nodal support through the sequencing of nuclear data in an order of magnitude three to five times greater than the size of GHR exon 10. As VLB applies the resampling of sites holding the same phylogenetic signal as GHR, this method may provide conservative predictions on the impact of further sequencing on the overall resolution of the arvicoline phylogenetic tree. By adding nuclear data sampled from different loci, we could expect an improvement in node resolution [67], in particular for nodes not well-supported by GHR data alone (e.g. nodes P, G). Since genetically independent loci have undergone different substitutional pressures, they may provide complementary resolution of the species tree, and increase the chance of finding molecular synapomorphies that resolve every node [13,29,57,64]. Furthermore, we predict that introns might be equally as efficient for retracing the phylogeny of voles and lemmings – as recently shown for similar recent evolutionary radiations [14,29,59] – because of their relaxed substitution constraints and their potentially greater number of informative sites.

## Conclusion

The nuclear exon 10 of the GHR performs better than the mitochondrial CYB for resolving Arvicolinae phylogenetic relationships. Support is found for a sister-group relationship between red-backed voles (Myodini) and a clade including water (*Arvicola*), snow (*Chionomys*), and meadow voles (*Microtus *and allies). Lemmings (*Lemmus *and *Dicrostonyx*) are found polyphyletic while the Caucasian long-clawed vole (*Prometheomys*) is among the basalmost arvicoline genera. Contrary to recent taxonomic suggestions, we do not obtain support for splitting *Blanfordimys*, *Lasiopodomys*, *Neodon*, and *Phaiomys *from *Microtus*. We concur with others [68-70] that the higher quality of nuclear genes resides in higher values of gamma parameter, uniform and stationary base compositions, and more uniform nucleotide substitution probabilities. The usefulness of nuclear exons for investigating 2–10 Myr old evolutionary histories is probably due to highly informative third codon positions, which keep a good compromise in their evolutionary rate: rapid enough to accumulate synapomorphies, yet slow enough to be not affected by substitutional saturation. The sequencing of nuclear DNA data in an order of magnitude three to five times greater than the size of GHR exon 10 might resolve most nodes of the Arvicolinae phylogeny provided that multiple genetically independent gene trees agree on the phylogenetic structure.

## Methods

### Taxonomic sampling and molecular biological methods

Our study is based on a total of 25 arvicoline species representing 14 of the 28 genera and 7 of the 10 tribes according to [33]. For the genus *Microtus*, we included 12 specimens belonging to different subgenera or species whose taxonomic status is questionable [35,36] (see Table 1). We used as outgroups, representatives of two lineages phylogenetically close to the Arvicolinae, *Mesocricetus auratus *(Cricetinae), and *Phyllotis darwinii *(Sigmodontinae) [42,71,30]. Two other taxa belonging to different subfamilies of Muridae, *Rattus norvegicus *(Murinae) and *Meriones *sp. (*M. shawi *+ *M. unguiculatus*; Gerbillinae), were chosen as more distant outgroups.

All ethanol-preserved arvicoline samples were stored in the mammalian tissue collection of the Institut des Sciences de l'Evolution de Montpellier [72]. Total DNA was extracted using the QIAamp DNA mini kit (Qiagen). Part of the GHR exon 10 was amplified and sequenced using the primers GHR5 forward (5' GGCRTTCATGAYAACTACAAACCTGACYTC 3') and GHR6 reverse (5' GAGGAGAGGAACCTTCTTTTTWTCAGGC 3'), and GHR3 forward (5' GACTTTATGCYCARGTRAG 3') and GHR4 reverse (5'-CTYACYTGRGCATAAAAGTC 3'). PCR conditions were 95°C 5 min, followed by 95°C 30 sec, 61°C 30 sec, 72°C 1 min (5 times), then 95°C 30 sec, 59°C 30 sec, 72°C 1 min (5 times), followed by 95°C 30 sec, 57°C 30 sec, 72°C 1 min (5 times), then 95°C 30 sec, 55°C 30 sec, 72°C 1 min (5 times), and then 95°C 30 sec, 53°C 30 sec, 72°C 1 min (20 times), with a final extension at 72°C 5 min.

The amplification and sequencing of the CYB were conducted using primers MVZ05 and MVZ14 [73] and additional internal ones MVZ16 [73] and H8 [74]. PCR products for GHR and CYB were purified from 1% agarose gels using Amicon Ultrafree-DNA columns (Millipore) and sequenced on both strands using automatic sequencing (Big Dye Terminator cycle kit) on an ABI 310 (PE Applied Biosystems).

All taxa included in our study were represented by both CYB and GHR sequences. A 921 bp segment of the exon 10 of GHR was sequenced for 22 specimens and the complete CYB (1140 bp) for 7 specimens. The arvicoline sequences new to this study have been deposited in the EMBL data bank, and we also used previously published sequences when available (see Table 1).

### Sequence alignment and phylogenetic analyses

Sequences were manually aligned with the ED editor of the MUST package [75]. Non-sequenced positions as well as introduced gaps were treated as missing data in subsequent analyses. Heterozygotic bases found in GHR sequences were coded following the IUPAC nucleotide ambiguity code.

Phylogenetic analyses of CYB and GHR alignments were conducted under the maximum likelihood (ML) and Bayesian methods, using PAUP* [76] version 4b10, PHYML [77] version 2.4.4, and MrBayes version 3.04 [78]. Moreover, MrBayes provided the opportunity to run analyses assuming different models of sequence evolution for each predefined partition, thus permitting the parameters estimated for each model of sequence evolution to be directly compared among codon positions and between genes. When CYB and GHR sequences were combined, sequences from different specimens of the same species were sometimes used. We assumed that the phylogeographic structure detected in some arvicoline species [45,79] was negligible as compared to the level of genetic divergence between the distinct *Microtus *subgenera and even arvicoline tribes here compared.

The program Modeltest [80] version 3.06, was used to determine the sequence evolution model that best fits our data using the Akaike Information Criterion (AIC). This program examined the fit of 56 models, with either a proportion of invariable sites (I), a gamma distribution of among-sites variation of substitution rates (Γ), or both (I + Γ). The best-fitting substitution models were TrN93 + Γ + I [81] for the GHR data set, and GTR + Γ + I [82] for the CYB data set. However, to run the same model of sequence evolution under PHYML and MrBayes, GTR was chosen for all phylogenetic analyses (the optimal GHR topology was not model-dependent, as it appeared that both GTR and TrN93 topologies were identical). To allow a fair comparison of α estimates of Γ-shape among genes and partitions, we did not use a proportion of invariable sites, but rather assigned eight discrete Γ categories (Γ_8_). The Γ distribution allows some sites to evolve at a very low rate, and the incorporation of a fraction of invariable sites does not necessarily lead to a significant increase in likelihood [83].

To avoid excessive calculation times, our PAUP* ML analyses were conducted in two steps. First, we estimated ML parameters on a neighbor-joining (NJ) starting tree. Second, a ML heuristic search was conducted by Tree Bisection Reconnection (TBR) branch swapping to identify the optimal tree under these constrained GTR + Γ_8 _parameter estimates. This tree was re-used for a new round of parameter estimation/branch swapping, and the procedure was repeated until there was a stabilization of both topologies and parameters. The stability of nodes was estimated by ML bootstrap percentages (BP_ML_) [84], computed by PHYML after 100 replicates of site resampling, with BioNJ starting trees. Because of its rapidity, PHYML was preferred over PAUP* for bootstrap analyses. To assess the amount of phylogenetic signal contained within an individual partition (each codon position of each gene), 500 replications of ML bootstrapping were also independently performed for each partition.

Bayesian analyses were performed with one distinct GTR + Γ_8 _model per gene and codon position, with unlinking base frequencies, GTR, and Γ parameters. Metropolis-Coupled Markov chain Monte Carlo (MCMCMC) sampling was conducted during 10,000,000 generations with five incrementally heated chains. We used Dirichlet priors for base frequencies (1,1,1,1) and for GTR parameters (1,1,1,1,1) scaled to the G-T rate, a Uniform(0.05,50.00) prior for the Γ shape, and an Exponential(10.0) prior for branch lengths. Bayesian posterior probabilities (PP) were computed from trees sampled every 100 generations, after removing the 50,000 first trees as the "burn-in" stage. In order to discriminate between moderately and strongly supported nodes – for which initial PP were superior to 0.95 – we also calculated bootstrapped Bayesian posterior probabilities (BP_bay_) as suggested by [85] and [86]. Due to computing time limitations, BP_bay _were only computed for the combined data set (GHR + CYB). First, 100 bootstrap pseudo-replicates were independently generated from each of the six partitions (the three CYB and the three GHR codon positions) using the SEQBOOT program 3.6a2.1 [87] of the PHYLIP package. Second, for each of the 100 concatenated bootstrap data sets, MCMCMC sampling of trees was performed as previously described for the original data under the six GTR + Γ_8 _partitioned model, except that trees were sampled every 100 generations for only 500,000 generations. To maximize the probability that the chains reached stationarity in each bootstrap replicate, one-half of the 5,000 trees sampled from the posterior probability distribution was systematically removed as the burnin [86]. BP_bay _resulted from the overall 50% majority rule consensus of the 500,000 saved trees.

Following [34], sequences were also analyzed at the amino-acid level. We respectively used the JTT + Γ + I and mtREV + Γ + I ML models of protein evolution for GHR and CYB sequences as implemented in PHYML.

### Sensitivity analyses

We performed sensitivity analyses using PAUP* to assess the relative contribution of the various model parameters to the log-likelihood increase when more complex models are considered. To take the evolutionary properties of each codon position into account, nucleotide sites were categorized into each of the three codon positions. The procedure was conducted separately for CYB and GHR sequences. All sets of parameters – base composition (BC), substitution rate matrix (GTR), heterogeneity of substitution rate among sites (Γ), and branch lengths (BL) – were estimated independently for each codon partition. AIC values were compared to assess the significance of likelihood variation between global and partitioned models. For instance, to test the contribution of BC, only BC parameters were computed for each partition whereas other parameter values (GTR, Γ, BL) were fixed for the whole gene.

### Evaluation of the saturation of nucleotide substitutions

The nucleotide saturation of the phylogenetic markers was assessed graphically according to the procedure of [88], by plotting the number of observed differences as a function of the ML inferred number of substitutions for all 351 pairwise comparisons for 27 sequences (the partial CYB sequence of *Lasiopodomys mandarinus *was removed). The inferred number of substitutions was estimated from the ML tree as the sum of the branch lengths linking two terminals. The level of saturation was estimated by the slope (S) of the linear regression between the observed and inferred substitutions. Substitutions saturation is evidenced when the number of inferred substitutions increased, whereas the number of observed differences remained constant. We performed saturation analyses independently for each phylogenetic marker and for each codon position. ML branch lengths were obtained for each partition using PAUP* and by enforcing the topology as identical to the combined CYB + GHR tree.

### Variable Length Bootstrap

To compare the phylogenetic resolving power of GHR and CYB at varying taxonomic levels, we used the variable-length bootstrap (VLB). In this method, bootstrap support is estimated as a function of a variable number of resampled characters [89]. For each data set, nucleotide sites were resampled to generate bootstrap pseudomatrices of 100, 250, and until 5000 characters with increasing steps of 250 sites. All bootstrap searches were then performed using ML analyses with PAUP*.

## Authors' contributions

TG and EJPD initiated the study, assembled the data, designed and ran the calculations. TG contributed to collect specimens in the field, obtained DNA sequences, and wrote the manuscript under the supervision of EJPD. MKT intensively helped TG in DNA sequencing and sequence alignment. EP initiated the project on arvicoline phylogenetics and obtained financial support. He contributed with TG to collect specimens and assisted with drafting. PC and SS provided CYB sequences and helped to improve the manuscript. All authors read and approved the final manuscript.

**Table 4:** Genera and species of Arvicolinae, with cytochrome *b *(CYB) and Growth Hormon Receptor (GHR) accession numbers and references.

**Subfamily (Genus)**	**(Subgenus) Species**	**Common name**	**CYB**	**CYB**	**GHR**	**GHR**	**Source**
			**Accession**	**Reference**	**Accession**	**Reference**	
Gerbillinae	*Meriones shawi*	Shaw's jird	-	-	AF332021	[90]	
	*Meriones unguiculatus*	Mongolian jird	AF159405	[91]	-	-	
Murinae	*Rattus norvegicus*	Brown Rat	VO1556	[92]	X16726	[93]	
Sigmodontinae	*Phyllotis darwini*	Darwin's leaf-eared mouse	U86819	[94]	AF332023	[90]	
Cricetinae	*Mesocricetus auratus*	Golden Hamster	AF119265	[34]	AF540632	[95]	
Arvicolinae							
*Arvicola*	*A. terrestris*	European water vole	AF119269	[34]	**AM392380**	This study	ISEM T-3054
*Blanfordimys*	*B. bucharicus*	Bucharian vole	**AM392369**	This study	**AM392392**	This study	ISEM T-1060
*Chionomys*	*C. nivalis*	European snow vole	**AM392367**	This study	**AM392378**	This study	ISEM T-523
*Dicrostonyx*	*D. torquatus*	Arctic Lemming	AF119275	[34]	**AM392381**	This study	ISEM T-1337
*Eothenomys*	*E. melanogaster*	Père David's vole	**AM392374**	This study	**AM392399**	This study	ISEM T-4338
*Lasiopodomys*	*L. mandarinus*	Mandarin vole	**AM392373**	This study	**AM392396**	This study	ISEM T-1066
*Lemmus*	*L. sibiricus*	Brown Lemming	AJ012671	[96]	**AM392398**	This study	ISEM T-1336
*Microtus*	*M. (Microtus) arvalis*	Common vole	U54488	[97]	**AM392386**	This study	ISEM T-3047
	*M. (Aulacomys) chrotorrhinus*	Rock vole	AF163893	[98]	**AM392383**	This study	ISEM T-603
	*M. (Aulacomys) richardsoni*	Water vole	AF163905	[98]	**AM392387**	This study	ISEM T-598
	*M. (Terricola) duodecimcostatus*	Mediterranean pine vole	**AM392375**	This study	**AM392400**	This study	ISEM T-4456
	*M. (Microtus) guentheri*	Gunther's vole	AY513804	[36]	**AM392397**	This study	ISEM T-4179
	*M. (Alexandromys) kikuchii*	Taiwan vole	AF163896	[98]	**AM392385**	This study	ISEM T-276
	*M. (?) longicaudus*	Long-tailed vole	AF119267	[34]	**AM392379**	This study	ISEM T-136
	*M. (Alexandromys) middendorffi*	Middendorf's vole	AF163898	[98]	**AM392390**	This study	ISEM T-3509
	*M. (Pedomys) ochrogaster*	Prairie vole	AF163901	[98]	**AM392389**	This study	ISEM T-130
	*M. (Alexandromys) oeconomus*	Tundra vole	AF163902	[98]	**AM392388**	This study	J.R. Michaux
	*M. (Mynomes) pennsylvanicus*	Meadow vole	AF119279	[34]	**AM392376**	This study	ISEM T-140
*Myodes*	*M. andersoni*	Japanese red-backed vole	AB037281	[99]	**AM392391**	This study	ISEM T-1341
	*M. glareolus*	Bank vole	**AM392368**	This study	**AM392384**	This study	ISEM T-1389
*Neodon*	*N. irene*	Chinese scrub vole	**AM392370**	This study	**AM392393**	This study	P. Giraudoux & J.-P. Quéré
*Ondatra*	*O. zibethicus*	Muskrat	AF119277	[34]	**AM392382**	This study	P.-A. Crochet
*Phaiomys*	*P. leucurus*	Blyth's vole	**AM392371**	This study	**AM392394**	This study	P. Giraudoux & J.-P. Quéré
*Phenacomys*	*P. intermedius*	Western heather vole	AF119260	[34]	**AM392377**	This study	ISEM T-672
*Prometheomys*	*P. schaposchnikowi*	Long-clawed mole-vole	**AM392372**	This study	**AM392395**	This study	ISEM T-377

When sequences are new for this study, accession numbers are in bold, and the source of the tissue is mentioned.
